# Ferroptosis plays a novel role in nonalcoholic steatohepatitis pathogenesis

**DOI:** 10.3389/fphar.2022.1055793

**Published:** 2022-12-02

**Authors:** Fei Xiong, Qiao Zhou, Xiaobo Huang, Peng Cao, Yi Wang

**Affiliations:** ^1^ Department of Gastroenterology, Sichuan Academy of Medical Science and Sichuan Provincial People’s Hospital, Chengdu, China; ^2^ Department of Rheumatology and Immunology, Sichuan Academy of Medical Science and Sichuan Provincial People’s Hospital, Chengdu, China; ^3^ Department of Critical Care Medicine, Sichuan Academy of Medical Science and Sichuan Provincial People’s Hospital, University of Electronic Science and Technology of China, Chengdu, China; ^4^ Department of Pharmacy, Union Hospital, Tongji Medical College, Huazhong University of Science and Technology, Wuhan, China

**Keywords:** ferroptosis, dihydroorotate dehydrogenase, mitochondrial glutathione, NASH, mitochondria

## Abstract

Ferroptosis relies on iron, and ferroptotic cell death is triggered when the balance of the oxidation-reduction system is disrupted by excessive lipid peroxide accumulation. A close relationship between ferroptosis and nonalcoholic steatohepatitis (NASH) is formed by phospholipid peroxidation substrates, bioactive iron, and reactive oxygen species (ROS) neutralization systems. Recent studies into ferroptosis during NASH development might reveal NASH pathogenesis and drug targets. Our review summarizes NASH pathogenesis from the perspective of ferroptosis mechanisms. Further, we discuss the relationship between mitochondrial dysfunction, ferroptosis, and NASH. Finally, potential pharmacological therapies directed to ferroptosis in NASH are hypothesized.

## Introduction

Nonalcoholic steatohepatitis (NASH) is a severe form of nonalcoholic fatty liver disease (NAFLD). Its histological characteristics are similar to those of alcoholic hepatitis, including macro-vesicular steatosis, hepatocyte ballooning, and necroinflammation, with or without fibrosis (Brunt et al., 1999; Matteoni et al., 1999; Kleiner et al., 2005). NASH may lead to cirrhosis in as many as 20% of patients (Matteoni et al., 1999) or to primary hepatocellular carcinoma (Angulo and Diehl, 2015; Wang et al., 2021). To date, lifestyle intervention is the immediate intervention for NASH (without fibrosis) (Diehl and Day, 2017), and pharmacological therapies for NASH are relatively rare, partly because NASH pathogenesis is too complex to be elucidated.

The liver is essential for regulating iron balance (Chen et al., 2022). Transferring receptor 1 (TFR1) and SLC39A14 deliver iron into hepatocytes and participate in many physiological and metabolic processes. Excess iron is stored as ferritin, and ferroportin (FPN) is critical for the elimination of iron. Hepcidin, ring finger protein 217, and a protein that regulates iron form a regulatory network that balances iron levels.

Similarly, some evidence suggests that iron plays a role in the progression of NASH (Bonkovsky et al., 1999; Facchini and Stoohs, 2002; Valenti et al., 2010; Nelson et al., 2011). In NASH patients, an increase in the haemochromatosis mutation rate is associated with an elevation in the hepatic iron level. More importantly, increased hepatic iron appears to be associated with liver fibrosis in NASH patients. Notably, iron is a crucial factor in insulin sensitivity. These studies imply that ferroptosis, a programmed cell death that relied on iron homeostasis, may play a role in the pathogenesis of NASH.

In this review, we focus on the potential mechanism of ferroptosis involved in the development of NASH and summarize some possible targets related to ferroptosis that may be important to NASH therapy.

## Ferroptosis, mitochondria, and nonalcoholic steatohepatitis

In 2012, Dixon, Lemberg (Dixon et al., 2012) discovered a unique mode of death mediated by erastin in cancerous cells. They also found that certain iron chelators inhibited this unusual death modality and concluded that the death modality relied on iron; therefore, they called this form of death “ferroptosis.” They further found that the target of erastin was the cystine-glutamate transporter X_C_^−^ and that ferroptosis was triggered after cystine-glutamate transporter X_C_^−^ activity was inhibited by erastin. Cysteine deprivation (Gao et al., 2015) or suppression of cystine-glutamate transporter X_C_^−^ disrupt glutathione (GSH) synthesis. GSH is an important antioxidant, and its depletion results in glutathione peroxidase 4 (GPX4) deactivation. Under normal conditions, hydroperoxides formed by PUFAs are reduced by GPX4. Inactivation of GPX4 leads to the accumulation of lipid peroxide. Bioactive iron triggers lipid peroxidation amplifies the generation of free oxygen radical species *via* the Fenton reaction and intensifies the dysregulation of oxidative lipid metabolism. Overall, the balance of the oxidation-reduction system in the cell is disrupted during ferroptosis. The mechanism map suggests a kind of strong connection between ferroptosis and diseases (Stockwell et al., 2017).

Because iron metabolism is closely associated with the liver, it is not surprising that ferroptosis has been recently found to play an essential role in hepatic injury (Wang et al., 2017; Chen et al., 2022). More interestingly, Minoru Tanaka et al. found ferroptosis was the first type of cell death *via* the choline-deficient, methionine-supplemented diet model and mixed lineage kinase domain-like protein (MLKL) knockout mice. The research hints ferroptosis is an important player in NAFLD conversion to NASH (Tsurusaki et al., 2019). Notably, three characteristics of NASH suggest ferroptosis is involved in the progression of NASH:

1) Lipid accumulation is the basis of hepatic steatosis. Excess lipid accumulation induces oxidative stress (Chen et al., 2020). Some reports have reported a high level of PUFAs in plasma samples taken from patients with NASH (Loomba et al., 2015), and PUFA intake increases the risk of NAFLD (Xie et al., 2021). These lines of evidence imply that PUFAs profoundly impact NASH development. The substrates for polyunsaturated fatty acid-containing phospholipids (PUFA-PLs) are necessary for ferroptosis (Lei et al., 2022). Oxidized phospholipid-induced inflammation promotes the progression of NASH (Sun et al., 2020). In summary, this body of evidence suggests that hepatocytes in NASH patients are likely to be enriched with PUFA-PLs, creating a precondition for ferroptosis.
2) The available evidence suggests that hepatic iron is involved in NASH. In physiological conditions, FPN is mainly expressed in the cytomembrane of Kupffer cells and hepatocytes around the portal vein (Drakesmith et al., 2015). Wang F et al. have shown FPN plays an important role in iron mobilization of hepatocyte and iron storage in macrophage in hepatocyte-specific FPN1 deletion mice (Zhang et al., 2012). Prolonged inflammation can produce more interleukin-6 (IL-6), and the binding of IL-6 to its receptor activates JAK/STAT pathway, resulting in STAT3 phosphorylation. Phosphorylated STAT3 enters the cell nucleus and activates the Hepcidin gene expression *via* locating the binding site in the Hepcidin promoter region (Camaschella et al., 2020). The combination of Hepcidin and FPN results in the change of the spatial configuration of FPN and leads to FPN degradation *via* ubiquitination and internalization (Billesbølle et al., 2020). Low expression of FPN inhibits iron release in macrophages and iron absorption in the duodenum (Donovan et al., 2005). There is evidence that IL-6 increases in NASH patients (Wieckowska et al., 2008) and STAT3 activation plays an important role in the fibrosis of NASH (Zhao et al., 2021). The molecular mechanism suggests that hepatic iron is associated with the development of NASH in a variety of pathological processes. Insulin resistance (Mendler et al., 1999) is directly associated with hepatic iron levels, and lower blood sugar decreases the iron concentration and inhibits iron transport. Other researchers have found a correlation between the hepatic iron level and the mutation rate of the haemochromatosis gene (HFE) (Bonkovsky et al., 1999). As a significant symbol in NASH development, an increase in hepatic iron has been related to the degree of hepatic fibrosis (George et al., 1998; Valenti et al., 2010). More importantly, elevated citrate has been detected in NAFLD; citrate indirectly produces free radicals *via* the Fenton reaction (van de Wier et al., 2013). The Fenton reaction can amplify the number of free oxygen radical species. Notably, bioactive iron is an essential component of ferroptosis, suggesting that ferroptosis promotes the progression of NASH *via* the Fenton reaction.
3) The cellular antioxidant ability is impaired. As mentioned above, the antioxidant ability in NASH is damaged or exhausted. The depletion of mGSH (Serviddio et al., 2008) is only one of many influences, and a lack of a variety of antioxidants has been found in NASH patients (Baskol et al., 2007), indicating that the ROS neutralization system in NASH was damaged. In recent years, researchers found that loss of Nrf2 is the key factor for the development of NASH (Xu et al., 2019). Nrf2 inhibits fatty acid biosynthesis by down-regulating ATP-citrate lyase, acetyl-CoA carboxylase 1, fatty acid synthase et al. (Yates et al., 2009; Kitteringham et al., 2010; Wu and Klaassen, 2011). The inhibiting action of Nrf2 on fatty acid biosynthesis might be attributed to Nrf2-mediated inhibition of the nuclear receptor liver X receptor–α gene (LXR-α) (Kay et al., 2011). More importantly, as a reducing agent, NADPH is necessary for biocatalysis and biotransformation of oxidized GSH by glutathione reductase. Nrf2 regulates NADPH biosynthesis with the help of malic enzyme and isocitrate dehydrogenase (Thimmulappa et al., 2002). Low expression of Nrf2 does not only promote the synthesis of fatty acids but also leads to the loss of reduced glutathione. Nrf^−/−^ mice also show a correlation between Nrf2 and immunoreactions (Itoh et al., 2004). As indicated above, the inhibition of the cystine-glutamate transporter X_C_^−^ induces ferroptosis. Studies have shown that cystine deprivation and glutaminolysis regulate ferroptosis (Gao et al., 2015). These studies reveal that ferroptosis is accompanied by the depletion of GSH. GSH is the primary antioxidant in the mitochondrial repair system, and its depletion shows that the cellular antioxidant ability is diminished after ferroptosis.

In summary, considering phospholipid peroxidation substrates, bioactive iron, and antioxidant system inhibition, ferroptosis may play an essential role in NASH development. Some indirect evidence supports this supposition; for example, haem oxygenase-1 blocked the progression of steatohepatitis (Wang et al., 2010). Moreover, recent research showed that haem oxygenase-1 induced ferroptosis mediated *via* mitochondrial factors (Wang et al., 2016). Further, the human genetic study brought home this point. Xingguo Liu et al. found that in two patients with mitochondrial DNA depletion syndrome (MDS), DGUOK mutant hepatocyte-like cells and hepatocyte organoids were more susceptible to iron overload-induced ferroptosis (Guo et al., 2021).

Some reports have shown that NASH usually exhausts mitochondrial glutathione (mGSH) by increasing the mitochondrial cholesterol level (Serviddio et al., 2008; Josekutty et al., 2013). GSH plays a crucial role in the antioxidative system, and its function is facilitated by its reduction and conjugation (Forman et al., 2009). The depletion of mGSH suggests that mitochondria have lost antioxidative capacity and that ROS levels have increased. Hence, mGSH is at least part of the second hit in the 2-s hit theory of NASH. GSH levels are generally lower in NAFLD patients than in healthy people (Kumar et al., 2013). Although the details of mGSH transport have not been thoroughly explained (Ribas et al., 2014), a lower level of cellular GSH exacerbates mGSH depletion; that is, changes in cellular GSH charge and concentration affect mGSH levels. Data have shown that glutamate is usually increased in NAFLD patients (Gaggini et al., 2018); therefore, a decrease in GSH may partly contribute to increased glutaminolysis (Du et al., 2020). Moreover, some researchers have indicated that the cysteine level is increased in NAFLD patients (Kalhan et al., 2011). In summary, impaired mitochondria indicate damage to the ROS neutralization system.

Hence, glutamine is a critical factor in ferroptosis, and glutaminolysis regulates ferroptosis (Gao et al., 2015); α-ketoglutarate is a product of glutaminolysis and is part of the tricarboxylic acid cycle; therefore, mitochondria and ferroptosis seem to be linked (He et al., 2020). As mentioned above, mitochondrial dysfunction is accompanied by NASH development. Abnormal mitochondrial ultrastructure has also been observed in ferroptosis (Dixon et al., 2012). Similar to that in NASH, a change in mitochondrial morphology has been found in hepatocytes undergoing ferroptosis (Wang et al., 2017). Mitochondria are the primary sources of ROS (Murphy, 2009); therefore, mitochondria likely produce ROS, increasing lipid peroxidation. During NASH development, the depletion of mGSH implies that a decreased GSH level (Serviddio et al., 2008) is a result of mGSH exhaustion in mitochondria but not factors outside mitochondria. mGSH exhaustion-related ferroptosis is reported in programmed cell death of cardiomyocyte (Jang et al., 2021). RSL3 induced ferroptosis of cardiomyocytes *via* the accumulation of adrenoyl-phosphatidylethamines (PEs). The decrease of dicarboxylate carrier (DIC) and oxoglutarate carrier (OGC) leads to the depletion of mGSH and increase ROS. The interaction of mGSH and GPX4 regulates the process of ferroptosis with the help of the accumulation of PEs. The depletion of mGSH suggested that NASH may have a similar mechanism in NASH development.

**FIGURE 1:**
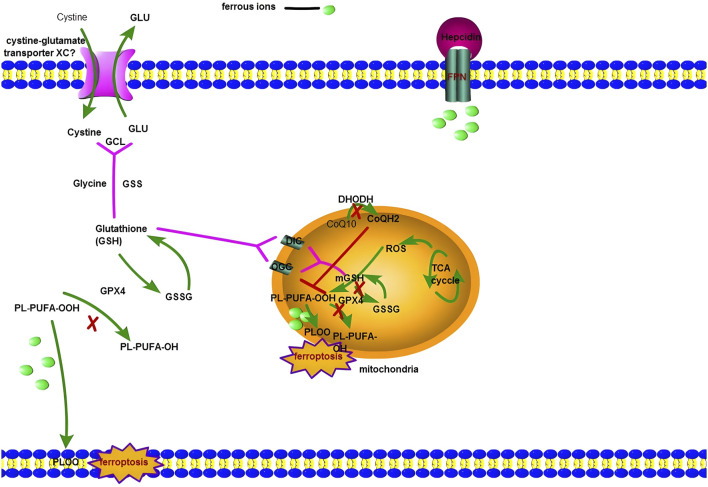
The figure shows ferroptosis involves both the cytoplasm and mitochondria. Cystine-glutamate transporter X_C_^−^ is responsible for the transfer of Cystine into the macrophage (or hepatocytes around the portal vein), then Cysteine takes part in the process of Glutathione biosynthesis. GPX4 regulates the reduced state (GSH) and oxidized state (GSSH). Meanwhile, PL-PUFA-OOH is converted to PL-PUFA-OH by GPX4. When prolonged inflammation can produce more interleukin-6 (IL-6), and the binding of IL-6 to its receptor activates hepatocyte’s JAK/STAT pathway, resulting in STAT3 phosphorylation. Phosphorylated STAT3 enters the hepatocyte nucleus and activates the Hepcidin gene expression by locating the binding site in the Hepcidin promoter region. A large amount of Hepcidin protein is finally secreted out of the cells. It results in the combination of Hepcidin and FPN and leads to FPN degradation *via* ubiquitination and internalization, so macrophages (or hepatocytes around the portal vein) accumulate more iron. When GPX4 is deactivated, the Fenton reaction can initiate ferroptosis in the cytoplasm. For NASH patients, there exists another mechanism: ferroptosis in mitochondria. Mitochondria have two kinds of antioxidase: GPX4 and DHODH. DHODH is located on the outside surface of the inner mitochondrial membrane. It can reduce coenzyme Q (CoQ) to ubiquinol (CoQH2). CoQ is a critical component in the electron transport chain. CoQH2 is the reduced form of CoQ; as an antioxidant, CoQH2 captures free radicals produced *via* lipid peroxidation. Hence, ferroptosis will start successfully in mitochondrial if DHODH and GPX4 are both deactivated.

Interestingly, the research showed the inhibition of DIC and OGC has a significant effect on mitochondrial membrane potential which was reduced by 14% less in PSA&BMA-treated cells (PSA, DIC inhibitor, and BMA, OGC inhibitor) than in the untreated cells (Jang et al., 2021). Earlier research also reported mitochondrial membrane potential changes in ferroptosis (Gao et al., 2019). The evolutionary formation of the membrane potential indicates that modification of membrane permeability—the transformation of membrane permeability—is most likely exacerbated by mGSH depletion. In the cellular defense mechanism, GPX4 is the most critical antioxidase; research has shown that lipid peroxidation induced by GPX4 is not evident in mitochondria (Angeli et al., 2014). However, further studies showed that GPX4 in mitochondria plays a defender in ferroptosis after dihydroorotate dehydrogenase (DHODH) is deactivated (Zhu et al., 2020; Mao et al., 2021). DHODH is located on the outside surface of the inner mitochondrial membrane. It can reduce coenzyme Q (CoQ) to ubiquinol (CoQH2). CoQ is a critical component in the electron transport chain. CoQH2 is the reduced form of CoQ; as an antioxidant, CoQH2 captures free radicals produced *via* lipid peroxidation. Hence, when the expression level of GPX4 is low, DHODH is expressed to prevent ferroptosis (Figure 1). Paradoxically, a recent study showed the benefit of using an inhibitor of DHODH to treat NAFLD (Zhu et al., 2020). Notably, the work concentrated on inflammatory cells, not hepatic cells or Kupffer cells. In summary, mitochondria are likely to regulate ferroptosis by inhibiting the ROS neutralization system.

## Potential drug targets of nonalcoholic steatohepatitis related to ferroptosis

To date, pharmacologic therapies for NASH have shown limited efficacy. As an antioxidant, vitamin E, which is used to treat NASH, has led to inconsistent results. A randomized control trial (RCT) showed that vitamin E or vitamin C reduced fibrosis scores (which was significant, *p* = 0.002) (Harrison et al., 2003). However, in another study, vitamin E treatment failed to reduce fibrosis scores (*p* = 0.24) (Sanyal et al., 2010). A meta-analysis revealed that trials with vitamin E indicated high heterogeneity (Musso et al., 2010). Some insulin sensitizers have shown a similar pattern (Musso et al., 2010). Furthermore, long-term use of vitamin E has a potential risk and large doses of vitamin E may increase all-cause mortality (Miller et al., 2005). In addition, vitamin E treatment may increase the risk of prostate cancer (Klein et al., 2011). More importantly, most trial periods are too short to maintain data accuracy, and these trials typically do not include crucial clinical outcomes, such as liver cirrhosis or liver cancer. Even the curative effects of GLP-1 receptor agonists are unclear (Newsome et al., 2021), indicating the need for additional data on GLP-1 receptor agonist trials.

Furthermore, GLP-1 receptor agonists are used only for patients with NASH and diabetes, and the scope of treatment is limited. In summary, the mechanism of insulin resistance is unlikely to be sufficiently similar to those of potential drug targets for NASH; therefore, we need to change our minds: ferroptosis may be a more analogous system. That is, based on the mechanism of ferroptosis, potential pharmacologic therapies can be classified into three attributional categories: regulating substrates for phospholipid peroxidation, iron chelating, and repairing the ROS neutralization system.

Substrates of phospholipid peroxidation can be regulated; for example, statins are a choice for regulating PUFAs (Nozue et al., 2013). However, data on statins as therapeutic drugs are insufficient to prove their benefit for NASH patients (Tomasiewicz et al, 2018). Paradoxically, omega-3 polyunsaturated fatty acids are beneficial to NAFLD patient (Masterton et al., 2010). However, PUFA-PLs are also found in normal cells. It is essential to maintain certain physiological functions, such as the cell membrane fluidity structure of the cytomembrane; therefore, PUFA-PLs may be necessary for hepatic cells. In addition, considering the mechanism, cells’ oxidation-reduction system is imbalanced during ferroptosis. When the imbalanced oxidation-reduction system cannot be corrected, the simple reduction in PUFAs may not be enough to inhibit ferroptosis. Therefore, regulating substrates for phospholipid peroxidation is not a key factor in ferroptosis.

Iron chelators appear to be good choices for regulating ferroptosis. Brent Stockwell et al. discovered ferroptosis was inhibited *via* the intervention of certain iron chelators (Dixon et al., 2012). However, in a cohort study, the main clinical effects of iron accumulation in NASH patients were insignificant (Younossi et al., 1999). In this unselected cohort, certain unknown confounding factors were not eliminated, and the number of patients with NASH was lower than that of the other cohort. Therefore, further research is needed. In the past, targeted pharmacologic therapies for NASH usually focused on insulin resistance and antioxidants; fewer studies have been directed to hepatic iron as a drug target for NASH. Notably, no significant benefit has been obtained *via* phlebotomy (Adams et al., 2015); hepatic iron was not adequately removed. In the clinic, a chelating agent is a potential therapeutic choice. For example, deferoxamine is usually suitable for beta-thalassemia and other iron-overload conditions. Compared with phlebotomy, hepatic parenchymal cells can take up more iron. This means that deferoxamine can clear iron directly from hepatocytes. Indeed, the half-life of deferoxamine is short; the potential question is whether iron accumulates in mitochondria. In rat hepatic cells, the iron level in mitochondria was double that of the cytoplasm (Paul et al., 2017). Iron chelators may not be able to capture enough bioactive iron in mitochondria to benefit NASH patients; therefore, further physiological efficacy and safety data need to be generated in future relevant clinical studies of NASH.

Repairing the ROS neutralization system *via* GSH is a good choice in ferroptosis. A pilot study showed a benefit from biochemical tests for patients with NASH (Irie et al., 2016). However, similar to iron chelators, GSH levels can be exhausted in mitochondria (Serviddio et al., 2008). It is unclear whether supplementation with GSH can lead to mGSH level recovery. A method to transport GSH into mitochondria is also a challenge. Additional data on GSH effects are needed in the future to develop mGSH-targeted pharmacologic therapy for NASH. We now know that GPX4 and DHODH are two central defense systems against ferroptosis in mitochondria (Mao et al., 2021). As DHODH is depleted, the defense system increasingly relies on GPX4 and *vice versa*. Pharmaceutical inhibitors of DHODH are potential for cancer treatment; however, the same drug target is not useful for developing NASH therapies.

Interestingly, a DHODH inhibitor drug, vidofludimus, showed the potential to reverse hepatic steatosis and reduce inflammation (Zhu et al., 2020). However, the results of these studies do not seem to comport with the mechanism of ferroptosis. Immune cells were the targets in the study. However, one hypothesis suggests that DHODH inhibition upregulates the expression of GPX4 under certain conditions and thus inhibits ferroptosis. In addition, a recent study of the regulation of cancer immunity by ferroptosis sheds some light on that question. Weiping Zou, Weimin Wang et al. found CD8^+^ cells induce tumor ferroptosis by interferon gamma they produced (Wang et al., 2019). The research first discovered ferroptosis is a new mechanism of anti-tumor, and it has widely applied prospects in the immunotherapy of tumours. According to this line of thinking, In the development of NASH, vidofludimus may regulate hepatic cell ferroptosis *via* the inhibition of Immune cells. Additional research is needed to explore the relevant mechanism of DHODH action in ferroptosis.

## Conclusion and perspectives

Importantly, although the pathogenesis of NASH is still debated, a large body of evidence shows that ferroptosis likely plays an essential role in NASH development. Evidence suggesting a relationship between ferroptosis and NASH has been mainly focused on three aspects: (Brunt et al., 1999) A high level of PUFAs is found in NASH patients, and PUFAs undergo oxidative phosphorylation, a process that induces inflammation. These characteristics suggest that PUFA-PLs play roles in NASH. (Kleiner et al., 2005) Iron participates in the development of NASH. Importantly, the Fenton reaction has been associated with the development of NASH; this reaction can amplify the number of free oxygen radical species involved in ferroptosis. (Matteoni et al., 1999) Evidence from a wide range of sources suggests that the balance of the oxidation-reduction system in hepatic cells is disrupted in NASH. Considering these three characteristics, we have discussed lipid-lowering agents, iron chelators, and GSH for use in drug therapies for NASH. Furthermore, we explored potential drug targets, such as bioactive iron in mitochondria and DHODH inhibitors.

As the research into ferroptosis in NASH advances, we believe significant progress will be made to show the future relationship between ferroptosis and NASH, providing new ideas and laying a foundation for identifying potential pharmacologic targets for NASH.

Interestingly, accumulating evidence suggests that mitochondria may play an important role in the progression of NASH and potentially in ferroptosis. Considerable evidence supports the idea that impaired mitochondrial function is involved in NASH, as indicated by abnormal morphology, gene expression, defective mitophagy, and depletion of mGSH. Similar evidence is found for ferroptosis. Thus, a strong association between NASH and ferroptosis seems possible. With intensive ongoing research, a better understanding of how mitochondria are involved in NASH and ferroptosis will contribute to resolving the debate on whether mitochondria regulate ferroptosis.
